# Efficacy and Safety of *Mycobacterium indicus pranii* as an adjunct therapy in Category II pulmonary tuberculosis in a randomized trial

**DOI:** 10.1038/s41598-017-03514-1

**Published:** 2017-06-13

**Authors:** Surendra K. Sharma, Kiran Katoch, Rohit Sarin, Raman Balambal, Nirmal Kumar Jain, Naresh Patel, Kolluri J. R. Murthy, Neeta Singla, P. K. Saha, Ashwani Khanna, Urvashi Singh, Sanjiv Kumar, A. Sengupta, J. N. Banavaliker, D. S. Chauhan, Shailendra Sachan, Mohammad Wasim, Sanjay Tripathi, Nilesh Dutt, Nitin Jain, Nalin Joshi, Sita Ram Raju Penmesta, Sumanlatha Gaddam, Sanjay Gupta, Bakulesh Khamar, Bindu Dey, Dipendra K. Mitra, Sunil K. Arora, Sangeeta Bhaskar, Rajni Rani

**Affiliations:** 1 All India Institute of Medical Sciences, New Delhi, India; 2 National JALMA Institute of Leprosy and Other Mycobacterial Diseases (ICMR), Agra, India; 3 National Institute of Tuberculosis and Respiratory Diseases, New Delhi, India; 4 National Institute of Research in Tuberculosis (ICMR), Chennai, India; 5 SMS Medical College (Hospital for Chest Diseases and TB), Jaipur, Rajasthan India; 6 NHL Municipal Medical College, Ahmadabad, Gujarat India; 7 Mahavir Hospital and Research Centre, Hyderabad, Andhra Pradesh India; 8 RBTB Hospital, New Delhi, India; 9 Catalyst Clinical Services Pvt. Ltd., New Delhi, India; 10 Cadila Pharmaceuticals Ltd., Ahmadabad, India; 11 Department of Biotechnology, New Delhi, India; 12 Post Graduate Institute of Medical Education & Research, Chandigarh, India; 13 National Institute of Immunology, New Delhi, India; 14 Chest Clinic and Hospital, New Delhi, India; 15 Systems Biology laboratory, CSIR-Institute of Genomics & Integrative Biology, New Delhi, India

## Abstract

Prolonged treatment of tuberculosis (TB) often leads to poor compliance, default and relapse, converting primary TB patients into category II TB (Cat IITB) cases, many of whom may convert to multi-drug resistant TB (MDR-TB). We have evaluated the immunotherapeutic potential of *Mycobacterium indicus pranii* (*MIP*) as an adjunct to Anti-Tubercular Treatment (ATT) in Cat II pulmonary TB (PTB) patients in a prospective, randomized, double blind, placebo controlled, multicentric clinical trial. 890 sputum smear positive Cat II PTB patients were randomized to receive either six intra-dermal injections (2 + 4) of heat-killed *MIP* at a dose of 5 × 10^8^ bacilli or placebo once in 2 weeks for 2 months. Sputum smear and culture examinations were performed at different time points. *MIP* was safe with no adverse effects. While sputum smear conversion did not show any statistically significant difference, significantly higher number of patients (67.1%) in the *MIP* group achieved sputum culture conversion at fourth week compared to the placebo (57%) group (p = 0.0002), suggesting a role of *MIP* in clearance of the bacilli. Since live bacteria are the major contributors for sustained incidence of TB, the potential of *MIP* in clearance of the bacilli has far reaching implications in controlling the spread of the disease.

## Introduction

Tuberculosis (TB) caused by *Mycobacterium tuberculosis* (*M*.*tb*) remains one of the major infectious diseases despite global efforts to contain and treat the disease which includes the Anti-Tubercular Treatment (ATT) through Directly Observed Treatment, Short-course (DOTS) strategy. Although, most of the *M*.*tb* infected individuals do not get the disease due to robust immune system, a substantial number progress to full-blown disease as soon as the immune system is compromised resulting in massive bacterial replication^1^. Despite good cure rate through ATT in primary TB, cases of default, non-compliance & relapse are rampant converting these patients into category II (Cat II) TB. Cat II TB includes those patients who have failed previous TB treatment, relapsed after treatment, or have defaulted during previous treatment^2^. A significant number of Cat II TB patients have been reported to develop MDR-TB^2^. Hence, an effective treatment for Cat II TB patients would help in containing the infection and prevent Cat II patients converting into MDR cases.

Immunotherapy as an adjunct to standard ATT could be an important strategy to treat this category of patients. In the present study, we have evaluated the immunotherapeutic potential of *Mycobacterium w*, renamed as *Mycobacterium indicus pranii* (*MIP*) in Cat II Pulmonary TB (Cat II PTB) patients. *MIP* shares antigens with both *M*. *leprae* and *M*.*tb* and has been shown to have significant protection against TB in both BCG responder and non-responder strains of mice^3–7^. *MIP* has been shown to have both immunotherapeutic and immunoprophylactic effects in multibacillary leprosy patients and their contacts respectively, in both hospital and population based trials^8, 9^. It induced conversion from lepromin negativity to lepromin positivity in multibacillary leprosy patients suggesting improved immune response to *M*. *leprae* antigens^10^. It also reduced the bacillary load, upgraded the lesions histopathologically, led to complete clearance of granuloma and reduced the duration of multi-drug therapy in leprosy patients^8^. In a large scale double blind trial for immunoprophylaxis against leprosy, involving 28,948 people in 272 villages of Ghatampur, Kanpur, India, a retrospective analysis after 13 years showed that incidence of TB was significantly reduced in the healthy contacts of leprosy patients vaccinated with *MIP* as compared to the placebo group^11^. Also, in a pilot study, addition of *MIP* to the short course chemotherapy in PTB patients led to earlier sputum conversion by at least 30 days^4^. Based on these encouraging results, we conducted a multi-centric trial to evaluate the safety and efficacy of *MIP* as an adjunct to ATT in Cat II PTB patients registered under the Revised National Tuberculosis Control Programme-Directly Observed Treatment, Short-course (RNTCP-DOTS).

## Results

**Figure 1:**
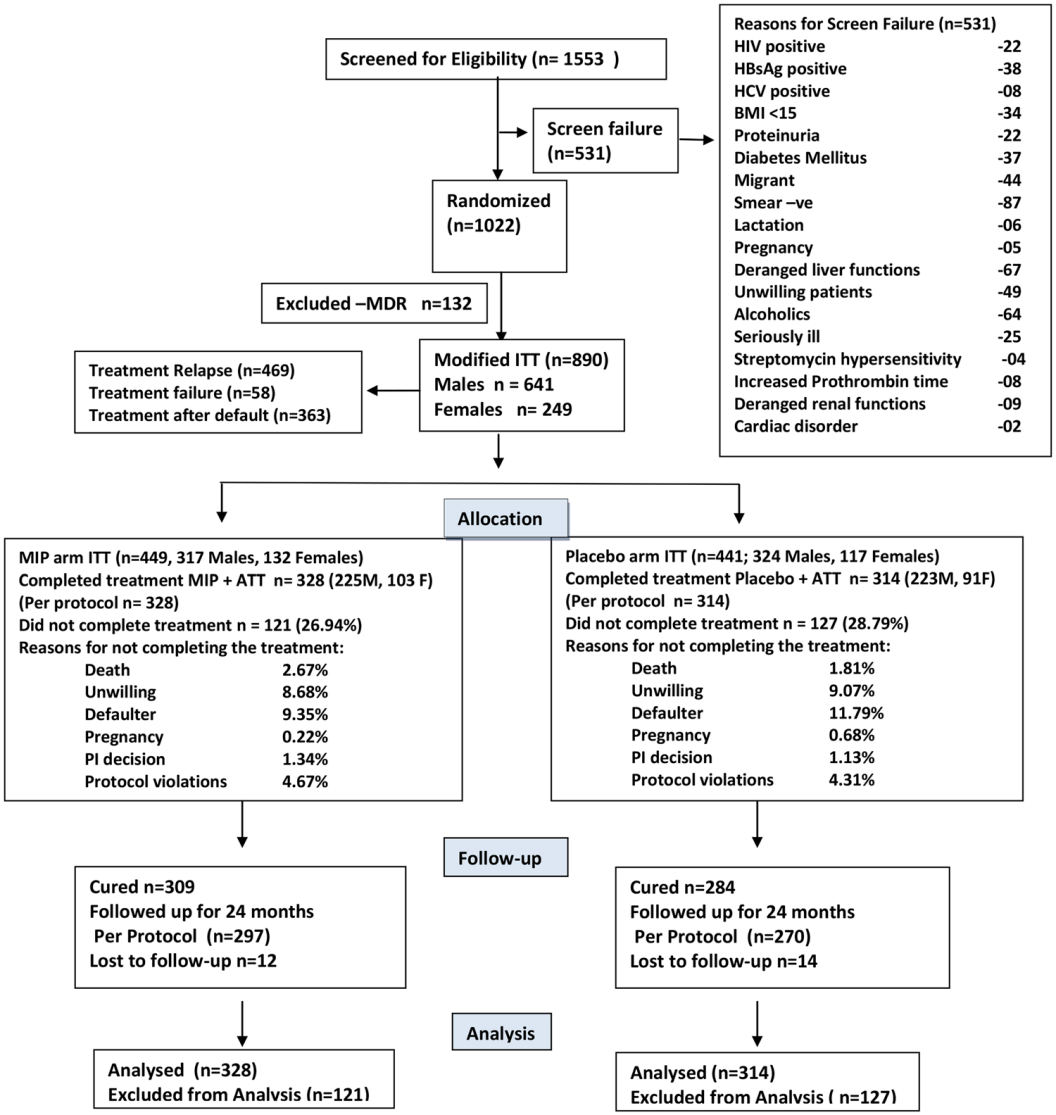
CONSORT Flow chart of Cat II patients screened, randomized, treated and analyzed. All randomized patients excluding MDRs (at baseline) formed the Modified intention to treat (ITT) population whereas all patients who have completed 8/9 months of study treatment (including 12 weeks of intensive phase) formed the per protocol population.

A total of 1553 Cat II PTB patients were screened of which 531 were screen failure and remaining 1022 were randomized in 1:1 ratio into the two treatment groups. However, 132 of these, later diagnosed with MDR tuberculosis (resistant to both rifampicin and isoniazid with or without any other drug), were excluded from the final analysis (Fig. 1). Among the 890 eligible patients (*MIP* Arm = 449, Placebo Arm = 441) that were included in the modified intent to treat (ITT), 642 (72.13%) (*MIP* Arm = 328, Placebo Arm = 314) patients completed the full course of treatment and were included in per protocol analysis (Fig. 1). 121 patients in the *MIP* arm (26.94%) and 127 patients in the Placebo arm (28.79%) did not complete the full course of treatment. There were multiple reasons for not completing the treatment which included death, unwillingness to continue in the trial, default in taking the treatment, pregnancy, protocol violations etc. (Supplementary Table 1 and Fig. 1). There was no significant difference in the baseline features of those who did not complete the treatment, in *MIP* and placebo group except that there were significantly higher number of cases with one drug resistance (p = 0.014) and significantly lower number of cases who had Sputum AFB smear grade of 2+ (p = 0.036) in the *MIP* group compared with placebo (Supplementary Table 2). 567 (297 in *MIP* arm and 270 in placebo arm) of the 642 cases who completed the treatment (88.32%), were followed up for two-years and were included in the relapse analysis. 26 patients were lost to follow-up for relapse analysis. There was no significant difference between the two arms in baseline characteristics such as, age, gender, BMI, sputum AFB grade, resistance to drugs, at initiation or radiographic severity (Table 1) in the patients who completed the treatment. *MIP* was safe and induced self-healing local reaction at the site of injection that subsided shortly.

**Table 1:** Distribution of patient characteristics at baseline for Modified intent to treat (ITT, N = 890) and Per Protocol (N = 642) population who completed the treatment.

Characteristic(s)	Modified ITT Population (N = 890)		Per Protocol Population (N = 642)	
	*MIP* Arm, (N = 449) n (%)	Placebo Arm, (N = 441) n (%)	*MIP* Arm, (N = 328) n (%)	Placebo Arm, (N = 314) n (%)
Age Group				
18–30 years	249 (55.5%)	224 (50.8%)	195 (59%)	166 (53%)
31–44 years	133 (29.6%)	154 (34.9%)	92(28%)	106 (34%)
≥45 years	67 (14.9%)	63 (14.3%)	41 (13%)	42 (13%)
Gender				
Male	317 (70.6%)	324 (73.5%)	225 (69%)	223 (71%)
Female	132 (29.4%)	117 (26.5%)	103 (31%)	91 (29%)
BMI*				
<18.5	441 (98.2%)	425 (96.4%)	322 (98%)	304 (97%)
≥18.5	8(1.8%)	16 (3.6%)	06 (2%)	10 (3%)
Reason for Inclusion in CAT-II				
Treatment Failure in CAT-I	34 (7.6%)	24 (5.4%)	23 (7%)	11 (4%)
Treatment after Default	189 (42.1%)	174 (39.5%)	129 (39%)	125 (40%)
Relapse	226 (50.3%)	243 (55.1%)	176 (54%)	178 (57%)
Resistance to Drugs at Treatment Initiation				
>3 drugs	0	0	0	0
2–3 drugs	41 (9.1%)	48 (10.9%)	16 (5%)	21 (7%)
1 drug	84 (18.7%)	60 (13.6%)	54 (16%)	44 (14%)
Streptomycin	40 (8.9%)	44 (10%)	24 (7%)	25 (8%)
Isoniazid	78 (17.4%)	80 (18.1%)	43 (13%)	48 (15%)
Rifampicin	15 (3.3%)	9 (2%)	08 (2%)	04 (1%)
Ethambutol	40 (8.9%)	30 (6.8%)	18 (5%)	12 (4%)
MDR#	63 (14%)	69 (15.6%)	—	—
Sputum AFB Smear Grade^@^				
1+	145 (32.3%)	122 (27.7%)	104 (32%)	83 (26%)
2+	117 (26.1%)	127 (28.8%)	93 (28%)	87 (28%)
3+	168 (37.4%)	170 (38.5%)	115 (35%)	128 (41%)
Sc	19 (4.2%)	22 (5%)	16 (5%)	16 (5%)
Chest Radiography at Treatment Initiation				
Bilateral cavitations	353 (78.6%)	336 (76.2%)	248 (76%)	233 (74%)
Unilateral cavitation	96 (21.4%)	105 (23.8%)	80 (24%)	81 (26%)
No cavitation	—	—	—	—
Radiographic Severity of Disease at				
Initiation^$^	54 (12%)	57 (12.9%)	40 (12%)	40 (13%)
Minimal	306 (68.2%)	304 (68.9%)	228 (70%)	220 (70%)
Moderately Advanced	88 (19.6%)	80 (18.1%)	60 (18%)	54 (17%)
Far Advanced				

*BMI- body mass index [weight in kg/ (height in cm)^2^]; ^#^MDR- Multidrug resistant tuberculosis ^@^WHO sputum grading - > 10 AFB/oil immersion field in at least 20 fields: 3+, 1–10 AFB/oil immersion field in at least 50 fields:2+, 10–99 AFB/ 100 oil immersion field:1+, 1–9 AFB/100 oil immersion field: Sc i.e., scanty);. ^$^Radiographic severity at baseline not available for 1 patient.

### Evaluation of the Primary outcome measures

#### The time of sputum conversion as well as comparison of early sputum conversion between the two groups

The primary outcome measures included time of sputum conversion. So, we studied the time of both sputum smear and sputum culture conversion, after administration of *MIP* or placebo along with ATT. Subjects who completed the full course of treatment were included in the data analysis and the time point at which the sputum smear or culture conversion took place was noted for each individual in both groups. The median time for sputum smear conversion was 2 weeks in both the groups. 53.35% patients in *MIP* group and 48.72% patients in placebo group turned sputum smear negative at 2 weeks and this difference was not significant statistically (Table 2). However, the median time for sputum culture conversion was 4 weeks after initiation of therapy in both the groups, suggesting that even when the cases became sputum smear negative, they were still harboring bacilli detectable in culture. Significantly higher number of patients (67.1%) in the *MIP* group achieved culture conversion at fourth week compared to the placebo (57%) group (p = 0.0002, OR = 1.86, 95% C.I. = 1.31–2.64) suggesting a role for *MIP* in eradication of detectable bacilli. This trend of significantly higher number of patients showing culture conversion in *MIP* group was observed at each subsequent visit after fourth week which indicates inability of conventional therapy to provide equivalent culture conversion (Table 2). By the time the follow-up reached 39^th^ week 94.2% of patients (309/328) in the *MIP* arm showed sputum culture conversion as compared to 89.17% (280/314) in the placebo arm. This difference was still significant even after correction of p value for multiple comparison (p = 0.02, OR = 1.97, 95% C.I.−1.06–3.75) (Table 2), suggesting the sustained effect of *MIP* in elimination of detectable bacilli.

**Table 2:** Sputum-smear and culture conversion time in per protocol group i.e. patients who completed the treatment.

Week	Smear conversion till (in weeks)				P	Odds Ratio (95% C.I.)	Culture conversion till (in weeks)				P	Odds Ratio (95% C.I.)
	*MIP* Arm (N = 328)		Placebo Arm(N = 314)				*MIP* Arm (N = 328)		Placebo Arm (N = 314)			
	Converted (Cumulative numbers)	%	Converted (Cumulative numbers)	%			Converted (Cumulative numbers)	%	Converted (Cumulative numbers)	%		
2	175	53.35	153	48.7	0.24	1.2 (0.87–1.66)	139	42.37	118	37.5	0.215	1.22 (0.87–1.69)
4	234	71.34	216	68.78	0.48	1.13 (0.79–1.61)	220	67.1	179	57.0	0.0002*	1.86 (1.31–2.64)
6	268	81.7	242	77.1	0.146	1.33 (0.89–1.98)	257	78.35	215	68.47	0.0046*	1.67 (1.15–2.41)
8	281	85.67	261	83.1	0.37	1.21 (0.77–1.9)	286	87.1	248	78.98	0.0054*	1.8 (1.16–2.84)
12	298	90.85	274	87.3	0.14	1.45 (0.85–2.48)	301	91.77	269	85.67	0.014*	1.86 (1.1–3.22)
16	303	92.37	280	89.17	0.16	1.47 (0.82–2.64)	303	92.37	270	85.98	0.009*	1.97 (1.14–3.46)
26	305	92.98	283	90.13	0.19	1.45 (0.79–2.67)	306	93.29	275	87.58	0.0136*	1.97 (1.1–3.58)
35	306	93.29	283	90.13	0.15	1.52 (0.83–2.83)	306	93.29	277	88.2	0.026**	1.86 (1.03–3.39)
36	—	—	—	—			309	94.2	280	89.17	0.02*	1.97 (1.06–3.75)
39	309	94.2	284	90.4	0.073	1.72 (0.91–3.3)	309	94.2	280	89.17	0.02*	1.97 (1.06–3.75)
40	—	—	—	—			—		281	89.49		
Median Time (range)	2 (2–39)		2 (2–39)				4 (2–36)		4 (2–40)			

*P value significant after Boneferroni’s correction. **Corrected p value not significant.

### Cure Rate

The cure rate in Cat II PTB patients was evaluated in both the groups after administration of *MIP* or Placebo as per RNTCP guidelines i.e. sputum smear conversion on at least two occasions, one of which was at the completion of treatment. The frequency of patients cured amongst those who completed the full course of treatment was 94.2% in the *MIP* group as compared to 90.4% in the placebo group at 39^th^ week after initiation of therapy (Table 2, sputum smear conversion and Supplementary Table 3), however, this difference was not statistically significant (p = 0.073, Odds Ratios (OR) = 1.72, 95% C.I. = 0.91–3.3). 68.8% of all patients (309 out of 449) who were initially inducted for the treatment (ITT) were cured in the *MIP* group as compared to 64.4% (284 out of 441) in the placebo group and this difference was also not statistically significant (p = 0.162, Supplementary Table 3).

### Relapse

Of the 642 patients 593 patients (*MIP* Arm = 309; Placebo Arm = 284) who were declared cured were eligible for relapse analysis. However, only 567 (*MIP* Arm = 297; Placebo Arm = 270) cases were analyzed for relapse as the data on relapse was not available for 26 patients. There was no significant difference in the relapse rate between the *MIP* and placebo groups at 6, 12, 18 and 24 months (Table 3).

**Table 3:** Relapse rate (n = 567)^‡^.

Interval (in months)	*MIP* Arm (N = 297)		Placebo Arm (N = 270)		p	Odds Ratio (95% C.I.)
	Relapsed	%	Relapsed	%		
6	23	7.74	16	5.92	0.39	1.33 (0.68–2.58)
12	6	2.02	7	2.59	0.64	0.77 (0.25–2.33)
18	2	0.67	2	0.74	0.65	0.9 (0.13–6.5)
24	3	1.01	1	0.37	0.34	2.7 (0.28–26.54)

^‡^Total eligible for Relapse Analysis = 593(*MIP* Arm = 309; Placebo Arm = 284). Among the 593 patients, data on relapse is not available for 26 patients as they were enrolled beyond the project timelines making the numbers available for evaluation of relapse rate as 567.

### Adverse Reactions

Cutaneous lesions and local reaction at the site of injection were observed in significantly higher number of patients in the *MIP* group compared to placebo group, as expected. However, these lesions were self-healing and subsided shortly. Non-vaccine related adverse events were significantly less in the *MIP* group as compared to placebo (p < 0.000035, Odds Ratio = 0.556) (Tables 4 and 5). The non-vaccine related adverse events included gastrointestinal, respiratory, CVS, skin or tuberculosis related events (Supplementary Table 4).

**Table 4:** Vaccine related adverse events.

Event Term	*MIP* Arm (N = 449)		Placebo Arm (N = 441)	
	Number of patients	Number of events	Number of patients	Number of events
Cutaneous Lesion at Injection Site (Abscess/Blister/Pappule)	309	310	10	10
Local Reaction at Injection Site (Pain, Swelling, Itching, Redness, Pus Discharge)	58	114	8	10
Scar at Injection Site	3	3	—	—
Total	370	427	18	20

**Table 5:** Non-Vaccine related adverse events.

Event Term	*MIP* Arm (N = 449)		Placebo Arm (N = 441)		P value	Odds Ratio (95% C.I.)
	Number of patients	Number of events	Number of patients	Number of events		
Total	127	159	183	217	0.000035*	0.556 (0.41–0.74)

*p value significant after Boneferroni’s correction.

### Post-hoc sub-group analysis

Since the cure was not observed in 100% of the patients in either *MIP* or placebo group, we further investigated whether the base line features of a patient were detrimental for the outcome of the treatments. While we realized during the analysis that sputum culture conversion should be considered as cure rather than sputum smear conversion, we did not deviate from our initial definition of cure i.e. sputum smear conversion on at least two occasions. So, further sub-group analysis was carried out based on sputum smear conversion only. To study the correlation, if any, between the baseline features of the patients and cure in the *MIP* and placebo groups, an analysis was carried out based on age, gender, BMI, reasons for inclusion, sputum bacillary load, and resistance to drugs at the baseline and radiographic severity (Table 6, Supplementary Table 5).

**Table 6:** Sub group analysis of baseline characteristics of cured patients-Per Protocol (those who completed the treatment).

Characteristic(s)	All patients in *MIP* Arm, (N = 328) n (%)	Cured in *MIP* Arm, (N = 309) n (% of the subgroup)	All patients in Placebo Arm, (N = 314) n (%)	Cured in Placebo Arm, (N = 284) n (% of the subgroup)	p Value	Odds Ratio	95% Confidence Interval
Age Group							
18–30 years	195 (59%)	186 (95.4%)	166 (53%)	152 (91.6%)	0.139	1.9	0.74–5.12
31–44 years	92(28%)	85 (92.4%)	106 (34%)	93 (87.7%)	0.278	1.7	0.6–5.3
≥45 years	41 (13%)	38 (92.7%)	42 (13%)	39 (92.9%)	0.65	0.97	0.33–2.9
Gender							
Male	225 (69%)	211 (93.8%)	223 (71%)	199 (89.2%)	0.085	1.8	0.87–3.91
Female	103 (31%)	98 (95.1%)	91 (29%)	85 (93.4%)	0.42	1.38	0.34–5.94
BMI							
<17.5	314 (95.7%)	295 (93.9%)	301 (95.8%)	271 (90.1%)	0.073	1.72	0.91–3.31
≥17.5	14 (4.3%)	14 (100%)	13 (4.2%)	13 (100%)			
BMI							
<18.5	322 (98%)	303 (94.1%)	304 (97%)	274 (90.1%)	0.065	1.75	0.93–3.36
≥18.5	06 (2%)	6 (100%)	10 (3%)	10 (100%)	—		
Reason for Inclusion in CAT-II							
Treatment Failure in CAT-I	23 (7%)	21 (91.3%)	11 (4%)	8 (72.7%)	0.17	3.54	0.99–12.71
Treatment after Default	129 (39%)	122 (94.6%)	125 (40%)	115 (92%)	0.41	1.52	0.5–4.85
Relapse	176 (54%)	166 (94.3%)	178 (57%)	161 (90.4%)	0.17	1.75	0.73–4.4
Resistance to Drugs at Treatment Initiation							
>3 drugs	0	0	0	0	0.046**	11.0	1.34–90.12
2–3 drugs	16 (5%)	16 (100%)	21 (7%)	16 (76.2%)	0.001*	25.38	3.3–194.9
1 drug	54 (16%)	54 (100%)	44 (14%)	36 (81.8%)	0.028**	6.35	1.71–23.59
Streptomycin	24 (7%)	23 (95.8%)	25 (8%)	18 (72%)	0.0653	3.48	1.22–9.88
Isoniazid	43 (13%)	41 (95.3%)	48 (15%)	40 (83.3%)	0.333	7.2	0.64–82.61
Rifampicin	08 (2%)	8 (100%)	04 (1%)	3 (75%)	0.152	8.8	0.96–80.5
Ethambutol	18 (5%)	18 (100%)	12 (4%)	10 (83.3%)			
Pyrazinamide		ND		ND			
Sputum AFB Smear Grade							
1+	104 (32%)	99 (95.2%)	83 (26%)	78 (93.9%)	0.713	1.26	0.28–5.72
2+	93 (28%)	87 (93.5%)	87 (28%)	83 (95.4%)	0.42	0.69	0.14–3.07
3+	115 (35%)	107 (93%)	128 (41%)	108 (84.4%)	0.035**	2.47	0.98–6.77
Sc	16 (5%)	16 (100%)	16 (5%)	15 (93.8%)	0.5	3.19	0.32–32.36
Chest Radiography at Treatment Initiation							
Bilateral cavitations	248 (76%)	235 (94.8%)	233 (74%)	208 (89.3%)	0.026**	2.17	1.04–4.74
Unilateral cavitations	80 (24%)	74 (92.5%)	81 (26%)	76 (93.8%)	0.49	0.81	0.18–3.34
No cavitations	—	—	—	—			
Radiographic Severity of Disease at Initiation^‡^							
Minimal	40 (12%)	40 (100%)	40 (13%)	38 (95%)	0.25	5.25	0.9–1.62
Moderately Advanced	228 (70%)	214 (93.9%)	220 (70%)	201 (91.4%)	0.31	1.44	0.21–23.36
Far Advanced	60 (18%)	55 (91.7%)	54 (17%)	45 (83.3%)	0.14	2.2	0.61–8.92

^‡^Radiographic severity at baseline not available for 1 patient; *p value Significant after Boneferroni’s correction. **Corrected p value not significant.

100% of the patients (16/16) who were resistant to two to three drugs were cured in *MIP* group as compared to 76.2% in the placebo group (16/21) and this difference was statistically significant (p = 0.046, OR = 11.0, 95% C.I. = 1.34–90.12). However, when each drug was analyzed separately, 95.8% of the patients who were resistant to streptomycin responded positively to *MIP* administration and were cured, compared to 72% in the placebo group (p = 0.028, OR = 6.35, 95% C.I. = 1.71–23.59). Similarly, 100% of the patients (54/54) who were resistant to one drug were cured in the *MIP* group as compared to 81.8% (36/44) in the placebo group (p = 0.001, OR = 25.38, 95% C.I. = 3.3–194.9).

*MIP* also had significant effect on patients with high bacillary loads. 93% of the patients (107/115) with a bacillary load of AFB3+ were cured in the *MIP* group compared to 84.4% (108/128) in the placebo group (p = 0.035, OR = 2.47, 95% C.I. = 0.98–6.77). Similarly 94.8% of the patients (235/248) with bilateral cavities were cured in the *MIP* group compared to 89.3% (208/233) in the placebo group (p = 0.026, OR 2.17, 95% C.I. = 1.04–4.74). These results highlight the benefits of *MIP* immunotherapy in the advanced cases of Cat II PTB who had drug resistance / higher bacillary indices / and had bilateral cavitations. However, these p values may not remain significant when corrected for multiple comparisons as the numbers of individuals involved in sub group analysis are small, suggesting that larger cohort of patients with drug resistance, higher bacillary indices and with bilateral cavitations need to be treated with *MIP* and evaluated before a conclusion can be drawn for its efficacy in ‘worse to treat’ Cat II TB.

### Immunological Parameters

PBMCs isolated from 57 blinded samples were studied for immunological parameters. When codes were opened, it was found that *MIP* group had 27 samples and placebo group had 30 samples.

There was a lot of heterogeneity observed in the samples studied for all the parameters at the base line and throughout the study period at one month, 3 months, 6 months and 12 months. Different assays done to assess the immune responses stimulated by *M*.*tb* and *MIP* antigens at different time points after vaccination / placebo administration did not show any significant differences between the two groups. This could be due to a very significant variance observed in all the groups studied at different time points as was evident from the scatter-graphs of all the assays (data not shown).

However, an important observation was that mean stimulation index on *in vitro* restimulation of PBMCs with *M*.*tb* antigen was consistently higher in the *MIP* group as compared to placebo group from 1 month till 6 month after the start of therapy. *MIP* group also showed significantly higher stimulation index (p < 0.047) as compared to healthy controls group at 3 months and 12 months (p < 0.013) when stimulated with *M*.*tb* antigen (Supplementary Figure 1). A subgroup analysis based on the initial bacterial index showed that patients with AFB3+ showed better proliferation indices at 1 and 3 months when given *MIP* as compared to placebo. Further stratification based on AFB3+ along with bilateral cavities showed higher proliferation indices at 1 month after administration of *MIP*.

The amount of IL-2 secreted after *in vitro* re stimulation with *M*.*tb* antigen showed an increasing trend in the *MIP* group from Day 0 to 6 month in follow-up samples on *M*.*tb* stimulation while no change was seen in the placebo group, however, the difference was not statistically significant (Supplementary Figure 2). Results of lymphocyte proliferation were also reflected in the IFN-γ secretion which increased consistently from Day 0 and almost doubled in 3 months and remained high till 12 month in the *MIP* group while on the contrary, the amount of secreted IFN-γ showed a decreasing trend in the placebo group from Day 0 onward till 6 month (Supplementary Figure 3). However, due to small number of samples in each group and also because of heterogeneity observed in the immune response of different patients at baseline itself differences observed between study group and placebo group were not statistically significant. Both IL-2 and IFN-γ showed an increase at the first month follow-up, in AFB3+ and AFB3+ along with bilateral cavities patients. Again, the difference was not statistically significant due to small number of cases in this subgroup and huge variance.

## Discussion

In spite of the continuous efforts made in reducing the burden of tuberculosis, the global epidemic of TB is growing, both due to untreated cases and rise in MDR-TB^12^. Prevalence of MDR-TB in Cat II TB patients has been reported to be high in various studies^2, 13^. Though there are clear WHO guidelines to treat Cat II patients, treatment failure rate reported in different studies range from 4% to 35%^14, 15^. These facts necessitate additional strategies to boost the immune responses in Cat II patients since they have the potential to become MDR or XDR (extensively drug resistant) TB cases. Addition of immunotherapy to ATT for Cat II PTB may be one such approach as it aims to restore the Th1/Th2 balance by enhancing the Th1 response and suppressing the excessive Th2 response^16^. Several approaches have been adopted, like use of anti-mycobacterial antibodies, use of mycobacterial antigens for boosting Th1 immune responses, administration of Th1 cytokines etc. (reviewed in detail by Uhlin *et al*.^17^). However, none were found suitable for large scale use.

An alternative approach is to use an immunomodulator which can induce multifaceted immune response and eliminate the tubercle bacilli with minimal side effects. Therapeutic and prophylactic effects of *M*. *vaccae* have been evaluated in a few studies. While *M*. *vaccae* was effective in preventing TB in PPD strong positive individuals, its immunotherapeutic effects were inconclusive^18–20^. While several new prophylactic vaccines are being evaluated to either replace BCG and/or as booster to BCG^21–25^, there is a dearth of studies on immunotherapeutic intervention along with ATT in TB patients. Immunotherapeutic potential of whole bacteria as well as individual specific antigens like hsp65^26^, Ag85B^27^, MPT-64, MPT-83^28^, DNA-acr, DNA-sod, Ag85C^29^ etc. have been evaluated in animal models of tuberculosis and had shown some promise but these subunit vaccines have their limitations for mass scale use in humans. Another candidate named RUTI that consists of liposome encapsulated detoxified bacterial fragments of *M*. *tuberculosis*, has undergone phase I and II trials on healthy participants and latently infected TB patients for safety, tolerability and antigenicity^30, 31^. However, there are very few studies on immunotherapy for treatment failure or category II TB patients.

A study reported that 62% of the treatment failure TB patients (n = 48) became sputum negative in one month when an oral vaccine called V-5 Immunitor (V5) was given to them^32^. However, in our study 91.3% (21/23) of the category I treatment failure patients were cured in the *MIP* group as compared to 72.7% (8/11) cases in placebo group (Table 4). Also, there is a serious limitation of this oral immunotherapy as the pill V-5 preparation is derived from pooled blood of HBV- and HCV-positive donors with chemical and heat inactivation^32^. Such formulation may not be practical for large scale use and has ethical issues due to the potential risk of developing HBV or HCV infections.

Our study is the first large scale, multi-centric trial on Cat II PTB where an immunomodulator *MIP* was given as an adjunct therapy along with standard ATT. Significantly higher number of patients in the *MIP* group showing sputum culture conversion as early as 4 weeks after initiation of therapy, suggests a role for *MIP* in elimination of detectable bacilli which may help in shortening the duration of treatment. While sputum smear conversion was envisaged as the primary read out for cure as per RNTCP guidelines, our data suggests that culture conversion may be a better marker for cure. At four weeks, while 68.78% of the cases in placebo group showed sputum smear conversion, only 57% of them showed sputum culture conversion (Table 2), suggesting that significantly higher number of cases (11.78%) in the placebo group were still harboring bacilli (p = 0.002, OR = 1.66, 95% C.I. = 1.19–2.3). On the other hand, in the *MIP* group, 71.34% of cases showed sputum smear conversion and 67.1% showed sputum culture conversion at fourth week and this difference was not significant statistically suggesting that most of the sputum smear negative cases had also become sputum culture negative in this group. Based on these data one can speculate that sputum smear conversion may actually be giving a false negative signal and one should trust sputum culture conversion to evaluate cure rate since the acid test for clearance of the detectable bacilli would be culture negativity. Support for this also comes from recent studies where sputum culture conversion has been considered as the only widely accepted marker of sterilizing activity and efficacy and used for monitoring therapy in patients with pulmonary TB which is crucial to avoid relapses^12, 33–35^. Time to culture conversion which effectively shows mycobacterial elimination, has been used in several trials to evaluate the efficacies of drugs like moxifloxacin, ciprofloxacin etc. in tuberculosis^35–38^. Perrin *et al*. also suggested that early or faster culture conversion could indeed be a better surrogate marker for effectiveness of new drug as compared to culture negativity at 2-month^38^.

Our post-hoc analysis showed a significant difference in cure rate in difficult to treat patients (with sputum AFB smear grade of 3+ and those with bilateral cavitations) in the *MIP* arm, which would definitely help in lowering the burden of TB. The immunological parameters also showed higher proliferation indices, IL-2 and IFN-gamma secretion in these cases given *MIP* compared to placebo. While calculating the sample size we had hoped to get at least 10% improvement over and above ATT, however, we saw 24% improvement in cases that were resistant to two to three drugs, where 100% of the patients were cured in the *MIP* group compared to only 76.2% in the placebo group. These advanced cases, otherwise, have poor prognosis and significant improvement in them provides rationale for inclusion of *MIP* in RNTCP. However, larger studies are needed to substantiate the findings of the present study.

*MIP* has been shown to have both immunotherapeutic as well as immunoprophylactic effects in multibacillary leprosy patients^8, 9^. While we could not correlate the immune responses with cure due to small sample size, *MIP* therapy as an adjunct to the chemotherapy in guinea pig model of tuberculosis accelerated bacterial killing through an increase in early protective Th1 immune response in the lungs of guinea pigs. This was followed by a balanced inflammatory and suppressive immune response in later part of the treatment suggesting that *MIP* induced immunomodulation is involved in accelerated bacterial clearance and reduction in the lung pathology^7^. Recent comparative genomic analysis of *MIP* has attributed its immunomodulatory properties to its high antigenic potential^39^.

Prophylactic potential of *MIP* against *M*.*tb* was earlier demonstrated in a retrospective population based double blind study in Ghatampur, India^9, 11^. However, the present study shows its clinical benefit as an immunotherapeutic agent for difficult to treat tuberculosis. At the time of initiation of the present study in 2005 the success rate of Cat II treatment in India was 68% compared to the success rate of 86% in newly sputum positive patients treated with Cat I treatment regimen. The retreatment outcomes for default and failed Cat I treatment varied from 48.8% to 55.8% in different studies^40^. However, in the present study, 94.2% of the cases in *MIP* group and 90.4% of the cases in the placebo group were cured at 39^th^ week. Higher cure even in the placebo group could be due to lower number of subjects enrolled with treatment failure (4%), treatment after default (40%) and removal of MDR patients from the study. Thus, this may be an effect of controlled conditions which is reflected in improved success rate.

Merits of the present study are that it is a large scale, multi-centric trial done meticulously and methodically with a good sample size. It is also the first study from India that has followed up patients after completion of treatment under Cat II regimen of the RNTCP. Shortcomings of the study are high number of cases (27.86%) who did not complete the treatment due to various reasons and heterogeneity in response rate across the study centers. Further, although there was slightly higher percentage of relapse in *MIP* group, the difference was not statistically significant from placebo group. The other drawback is small number of cases in the post-hoc subgroup analysis, suggesting that a much larger cohort needs to be evaluated for the efficacy of *MIP* in different sub-groups. Although the study was designed to be double blind, the cutaneous lesions and local reaction at the site of injection could have revealed the identity of the treatment to the doctors administering *MIP* or placebo. However, coded samples were sent to the laboratories for sputum smear and culture conversion and the laboratory staff involved in these analyses were totally blind to the treatment given to the patients. In spite of these short comings, our study clearly demonstrates superiority of *MIP-* immunomodulator in inducing early culture negativity and improving the cure rate of Cat II PTB patients with parameters that would include them in ‘difficult to treat’ category (like resistant to two or three drugs, and/or very high bacillary loads and/or bilateral cavitations) where current therapy is suboptimal and adjunct immunotherapy could assist in bacillary clearance. More studies in such cases would help strengthen the findings of the present study and justify inclusion of *MIP* in the RNTCP.

## Methods

### Study design and population

The study was a prospective, randomized, double blind, placebo controlled, multi-centric clinical trial. Patients aged between 18 to 60 years who were sputum smear positive and candidates for Cat II TB treatment under the RNTCP - DOTS were included in the trial after obtaining informed written consent. Cat II PTB patients included those with treatment after default (TAD), treatment failure (TF), and treatment relapse (TR). The study was approved by the Drugs Controller General of India (DCGI) in addition to the Institutional Ethics Committee at each site and was conducted adopting the ethical principles stated in the latest version of Helsinki Declaration as well as the applicable guidelines for good clinical practice (GCP). Ethical approvals were obtained from Institutional Ethics Committee of All India Institute of Medical Sciences, New Delhi, Ethical Committee of Central JALMA Institute of Leprosy, Agra, UP, Ethical Committee of Lala Ram Sarup Institute of Tuberculosis and Respiratory diseases, New Delhi, Ethics Committee of Bhagwan Mahavir Medical Research Centre, Hyderabad, Institutional Ethics Committee, NHL Municipal Medical College, Ahemadabad, Gujrat, Institutional Ethics Committee, Tubeculosis Research Centre (ICMR), Chennai, and Ethics Committee of SMS Medical College & Attached Hospitals, Jaipur. An independent Data Safety Monitoring Board (DSMB) was constituted to look at the safety aspects of the trial. The clinical trial was registered at the US National Institutes of health ClinicalTrials.gov Identifier: NCT00265226 available at their website (https://clinicaltrials.gov/ct2/show/NCT00265226).

Exclusion criteria included patients with extra-pulmonary tuberculosis, immunodeficiency, significant organ dysfunction (cardiac, renal or hepatic), co-infected with Hepatitis B or C virus, receiving cytotoxic therapy and pregnant or lactating women. Additional exclusion criteria were known hypersensitivity to ATT, seizure disorder, abnormal hematologic function and patients on oral and systemic steroids continuously for more than seven days, a week prior to inclusion. Patients with other serious or uncontrolled concurrent illness or with history of alcohol and drug abuse were also excluded. Eligible patients who consented to participate in the study were enrolled from 7^th^ March 2005 to February 2009. Primary completion date for data collection for primary outcome was December 31^st^, 2010 and follow up for 2 years after the completion of treatment finished in March 2011. The calculation of sample size was done assuming at least 10% difference in the sputum conversion rate at the end of active treatment as well as in the rate of relapse at 6, 12, 18 and 24 months of follow up period in the group getting *MIP* as an adjunct to ATT as compared to placebo. At 95% confidence interval and at power of 90, anticipating about 30% loss to follow up, at least 510 patients were required per group. The sample size was calculated as described in supplementary materials.

### Study protocol, Randomization and treatment plan

Eligible patients who consented to participate in the study were enrolled from 7^th^ March 2005 to February 2009 at seven centers in different parts of India, involved in tuberculosis research and treatment. 1553 patients were screened for exclusion and inclusion criteria and 1022 of them were included in the trial (Fig. 1). A randomization list was generated by an independent statistician using center-wise stratification with fixed sizes of blocks using Ralloc software. For every subject labeling of the vials was done as numbers (which had combination of centre number and the serial number). Double blinding was ensured by assigning unique numbers to each trial patient. Neither the investigators nor the patients were aware about the intervention they had received. The codes were kept with a person who had no conflict of interest with the trial. Codes were opened after final analysis duly approved by the trial DSMB.

Patients were randomized to receive either six intradermal injections of *MIP* at a dose of 5 × 10^8^ heat-killed bacilli in 0.1 ml normal saline or placebo (normal saline without bacilli) once in 2 weeks over 2 months. The schedule of administration of *MIP*/placebo was two simultaneous injections initially in the deltoid regions of both upper arms followed by single injection on alternate deltoid every two weeks, along with the standard chemotherapy, as per RNTCP guidelines. For the intensive phase, the patients received Streptomycin 750 mg (intramuscular injection) along with Isoniazid 600 mg + Rifampicin 450 mg + Ethambutol 1200 mg and Pyrazinamide 1500 mg (SHRZE i.e., S – Streptomycin, H- Isoniazid, R – Rifampicin, E – Ethambutol, Z – Pyrazinamide) orally three times in a week for two months followed by the same treatment but without Streptomycin i.e., HRZE for one month (Intensive Phase). If the patient was found to be sputum positive at the end of three months, the same treatment i.e., HRZE was continued for one more month. During the Intensive phase, the patients were administered the drugs during their visit at the clinic/hospital only. The intensive phase was followed by the continuous phase wherein the patients were administered Isoniazid 600 mg + Rifampicin 450 mg + Ethambutol 1200 mg three times in a week for five months. During the continuous phase the patients were dispensed medication on weekly basis from the study center.

Since the time of sputum conversion as well as the early sputum conversion between the 2 groups were to be compared, we performed both Sputum smear and sputum culture examinations at baseline and at weeks 2, 4, 6, 8, 12, 16, 26, 35, 36 and 39. In patients where the sputum smear was still positive at 3 months after initiation of the therapy, intensive phase was extended for one more month and further sputum examination was performed at 4, 6, 9 months of the treatment. Drug sensitivity was performed at baseline and at 3^rd^/4^th^ month. The patients were followed up at months 6, 12, 18 and 24 after the completion of the therapy.

### Primary Outcome Measures as defined in the registered trial NCT00265226 were as follows

The time of sputum conversion as well as the early sputum conversion between the 2 groups.

- The cure rate to be evaluated as the primary parameter of efficacy.
- The relapse in patients of category II tuberculosis to be compared in both the groups at an interval of 6, 12, 18 and 24 months after the completion of the therapy.
- Recording of any clinical adverse reactions at anytime during the study for assessment of safety.

### Study definitions

#### Cured

Initially sputum smear positive patient who had completed treatment and had negative sputum smears, on at least two occasions, one of which was at the completion of treatment.

#### Speed of Sputum Smear/Culture Conversion

The time-point at which first smear/culture turned negative for initially smear/culture positive patient who had completed treatment.

#### Relapse

A patient declared cured of TB but found to be bacteriologically positive during the 24 months follow-up period.

#### Definition of ITT and Per Protocol Population

All randomized patients excluding MDRs (at baseline) formed the Modified intention to treat (ITT) population whereas all patients who had completed 8/9 months of study treatment (including 12 weeks of intensive phase) formed the per protocol population.

### Sputum Smear Examination

Sputum smears were examined by Ziehl-Neelsen Staining (ZN) method for Acid-fast bacilli (AFB) using carbolfuchsin and methylene blue, as per the standards laid down under RNTCP guidelines. A grading of 3+ was assigned for more than 10 AFB per oil immersion field in at least 20 fields whereas 2+ and 1+ was assigned for 1–10 AFB per oil immersion field in at least 50 fields and 10–99 AFB per 100 oil immersion fields in at least 100 fields, respectively. A grading of scanty was assigned for 1–9 AFB per 100 oil immersion fields in at least 200 fields whereas negative was assigned for no AFB in 100 oil immersion fields in at least 100 fields.

### Sputum Culture Examination

AFB culture and sensitivity test were done using the Gold Standard Isolation Techniques: Lowenstein Jensen’s medium, radiometric or fluorescence method in different laboratories, as described in supplementary materials.

### Immunological parameters

To Study and compare the immunomodulatory effect of MIP in the study group as compared to placebo group immunological markers were studied in the peripheral blood of small representative group. Blood samples from 57 category II patients and 36 healthy controls were studied for immunological parameters. Blinded blood samples were collected at AIIMS at baseline, 1, 3, 6 and 12 month after treatment. Memory recall response was studied by lympho proliferation induced by *M*.*tb* and *MIP* sonicates using thymidine uptake assay. IFN-gamma, IL-12 and IL-4 production were studied using ELISPOT assays, Intracellular cytokines in CD4+ and CD8+ T cell compartments were determined by FACS after stimulation of PBMCs culture with sonicates of *M*.*tb* and *MIP*. The secreted cytokines were studied in the culture supernatants, which were stored frozen and assayed together using multiplex Cytometric Bead array system (human Th1/Th2 CBA kit) of BD Bioscience (USA) which included IL-2, IL-4, IL-5, IL-10, TNF-α, and IFN-γ. Unpaired t-test with Welch’s correction was used to analyze the significance of differences between the two groups with Graph-pad prism 4 software (San Diego, CA, USA).

### Statistical analysis

The data analysis was performed using Stata 9.2 program for windows. The primary efficacy end point was taken as per protocol principle and included patients who had completed 8/9 months of active treatment. In addition, a modified intent-to-treat (ITT) efficacy analysis was also performed. However, for safety analysis all patients were included as per the ICH-GCP guidelines. Chi-square test was used to compare baseline characteristics and cure rates between the two groups. Fisher’s exact test was used whenever the numbers were ≤5 in any group and one-tail analysis was used to calculate the p values. In such cases Odds ratios were calculated using Woolf’s method with Haldane’s modification^41^. p value of <0.05 was considered as statistically significant. Boneferroni’s correction was done by multiplying the p value by the number of comparisons done for each feature.

## Electronic supplementary material

Supplementary Materials
