# Supplementary material for: Efficacy and Safety of Mycobacterium indicus pranii as an adjunct therapy in Category II pulmonary tuberculosis in a randomized trial
Source: Sci Rep. 2017 Jun 13;7:3354. doi: 10.1038/s41598-017-03514-1 (PMC5469738; doi:10.1038/s41598-017-03514-1)
Supplement: Supplementary file 1 — Supplementary Materials [file 41598_2017_3514_MOESM1_ESM.pdf]

Efficacy and Safety of *Mycobacterium indicus pranii* as an adjunct therapy in Category II pulmonary tuberculosis in a randomized trial.

Surendra K Sharma[1*], Kiran Katoch[2], Rohit Sarin[3], Raman Balambal[4], Nirmal Kumar Jain[5], Naresh Patel[6], Kolluri J R Murthy[7], Neeta Singla[3], P K Saha[1], Ashwani Khanna[1], Urvashi Singh[1], Sanjiv Kumar[1], A. Sengupta[1,14], J N Banavaliker[8], D. S. Chauhan[2], Shailendra Sachan[2], Mohammad Wasim[2], Sanjay Tripathi[6], Nilesh Dutt[6], Nitin Jain[5], Nalin Joshi[5],Sita Ram Raju Penmesta[7], Sumanlatha Gaddam[7], Sanjay Gupta[9], Bakulesh Khamar[10], Bindu Dey[11], Dipendra K Mitra[1], Sunil Arora[12], Sangeeta Bhaskar[13], Rajni Rani[13,15*],

1. All India Institute of Medical Sciences, New Delhi, India
2. National JALMA Institute of Leprosy and Other Mycobacterial Diseases (ICMR) , Agra, India
3. National Institute of Tuberculosis and Respiratory Diseases, New Delhi, India
4. National Tuberculosis Research Institute (ICMR), Chennai, India
5. SMS Medical College (Hospital for Chest Diseases and TB), Jaipur, Rajasthan, India
6. NHL Municipal Medical College, Ahmadabad, Gujarat, India
7. Mahavir Hospital and Research Centre, Hyderabad, Andhra Pradesh, India
8. RBTB Hospital, New Delhi, India
9. Catalyst Clinical Services Pvt. Ltd., New Delhi, India
10. Cadila Pharmaceuticals Ltd., Ahmadabad, India
11. Department of Biotechnology, New Delhi, India
12. Post Graduate Institute of Medical Research, Chandigarh, India
13. National Institute of Immunology, New Delhi, India
14. Chest Clinic and Hospital, New Delhi
15. Current address: Systems Biology laboratory, CSIR-Institute of Genomics & Integrative Biology, New Delhi, India

Supplementary Materials

Supplementary Materials and Methods

Statistical Calculation for determining the sample size

Null Hypothesis:

Success rate with ATT = Success rate with ATT + *MIP* (*Mw*) vaccine

Alternate Hypothesis:

1. Success rate with ATT < Success rate with ATT + *MIP* (*Mw*) (1-sided)
2. Success rate with ATT ≠ Success rate with ATT + *MIP* (*Mw*) (2-sided)

Second alternate hypothesis seems implausible, because it is not possible for the success rate in ATT + *MIP* to be lower than ATT alone.

$$n = \frac{\{Z_{1-\alpha}.\sqrt{2P.(1-P)} + Z_{1-\beta}.\sqrt{P_1.(1-P)_1 + P_2(1-P_2)}\}^2}{(P_1 - P_2)^2}$$

Where; n = number of patients in each group

$Z_{1-\alpha}$ = Standard normal variate corresponding to one sided 5% level of significance (= 1.64)

$Z_{1-\beta}$ = Standard normal variate corresponding to 90% power (=1.282)

P_1 = Success rate with ATT in Cat II patients (=70%)

P_2 = Success rate with ATT + *MIP* vaccine in Cat II patients (80%)

P = $(P_1 + P_2)/2$ i.e. 75%

$$n = \frac{\{1.64..\sqrt{2(.75).(1-.75)} + 1.282\ .\sqrt{(.70).(1-.7) + (.8)(1-.8)}\}^2}{(.7-.8)^2}$$

= 320 patients per group

Anticipating about 30% loss to follow up, we required at least 320 X 1.3= 416 patients per group. If we consider two sided test then with 30% loss to follow up, we would have required at least 510 patients per group.

Sputum Smear Examination

Sputum smears were examined by Ziehl-Neelsen Staining (ZN) method for Acid-fast bacilli (AFB) using carbolfuchsin and methylene blue, as per the standards laid down under RNTCP guidelines. A grading of 3+ was assigned for more than 10 AFB per oil immersion field in at least 20 fields whereas 2+ and 1+ was assigned for 1-10 AFB per oil immersion field in at least 50 fields and 10-99 AFB per 100 oil immersion fields in at least 100 fields, respectively. A grading of scanty was assigned for 1-9 AFB per 100 oil immersion fields in at least 200 fields whereas negative was assigned for no AFB in 100 oil immersion fields in at least 100 fields.

Sputum Culture Examination

AFB culture and sensitivity test were done using the Gold Standard Isolation Techniques: Lowenstein Jensen's medium, radiometric or fluorescence method in different laboratories, as described in supplementary materials.

Collection of samples:

Sputum specimens from suspected TB patients were collected from each patient in a wide-mouthed screw capped containers [1].

Decontamination of the samples:

Samples were processed by modified Petroff's method (CDC). The samples were decontaminated by NALC/NaOH method. The specimens were incubated for 15 minutes with

equal volume of NALC/NaOH solution followed by neutralization with PBS (phosphate buffer saline). After centrifugation at 3000rpm for 30 min, supernatant was discarded and pellet re-suspended in 2ml of PBS.

Microscopy:

Decontaminated samples were stained by ZN technique. Concentrated sediment was spread over 2-3cm area on a clean glass slide. After heat fixing, it was flooded with hot Carbol Fuschin and left for 5 min. After washing, smear was decolorized with acid alcohol (3:97). Smear was then counterstained with methylene blue for 3min, and examined under an oil immersion microscope.

Isolation of *Mycobacterium tuberculosis* culture by Lowenstein Jensen's (LJ) medium:

Primary culture and first-line DST on LJ with final reading at 6 weeks were performed using standard methods, as previously described [2,3].

Isolation of *Mycobacterium tuberculosis* culture by Bactec 460 (Radiometric method)

Concentrated sediment was inoculated in BACTEC 12B medium and incubated at 37^0C (BACTEC TB medium (12B) is an enriched Middlebrook 7H9 base). This medium contains ^{14}C-labeled substrate. Mycobacteria utilize this substrate and release ^{14}CO2 into the atmosphere above the medium. When the vials are tested on the BACTEC 460 TB system instrument, the gas is aspirated from the vial and the ^{14}CO2 radioactivity is determined quantitatively in terms of numbers on a scale from 0-999. These numbers are designed as growth index (GI). Susceptibility and resistance were judged by comparison of the change in

the growth index of the control with that of the test drug as recommended. This interpretation gives susceptibility results at the 1% proportion basis [4].

Drug susceptibility Testing (DST):

Stock drug solutions were made as follows; Ethambutol, Streptomycin, Isoniazid, were dissolved in sterile double distilled water (DDW). Rifampicin was dissolved in N, N-Dimethylformamide (DMFO). These stock solutions were filter-sterilized and stored frozen at -20°C. Before the drug susceptibility testing, a tube of each stock solution of drug was thawed and diluted to the requisite concentration; added to each 7H12 vial with a tuberculin syringe, one vial being used for each drug. Thus, for each test a total of 11 vials were used, including one vial without drug to serve as a control. 0.1 ml of culture was inoculated into each of the drug-containing vial. For the control inoculum, 0.1 ml of this suspension was added to 9.9 ml of diluent (DDW). After thorough mixing, 0.1 ml of this was inoculated into the control vial. The inoculation of this 1:100 dilution of the suspension into the control was done to determine the conventional 1% level of resistance by comparing the growth in the control and drug-containing vials. All of the inoculated vials were incubated at 37°C and were checked daily with a BACTEC 460 instrument. A 5% CO_2-in-air mixture was used as the flushing gas for the bottles during each testing.

When an anti-tubercular drug is present in the medium, growth is inhibited if the mycobacteria are drug susceptible, resulting in the suppression of $^{14}CO_2$ production. The vials were read daily, at least 4-6 days. When the control GI reading was 30 or more, the results were interpreted by calculating the increase in GI from the previous day. If the difference in increase of GI of the control was greater than the difference in GI increase of the drug bottle,

the test mycobacteria were reported as susceptible to that drug. If the daily increase of GI of the control was less than that of the drug vial, the organisms were reported as resistant to that drug. Since the size of the inoculum in the control was 1/100 of the inoculum for the drug vial, the critical proportion for resistance was ascertained at the 1% level by comparing the growth rate of the control and drug vials.

Drugs & MICs:

S.No.	Drug	MIC
1	Rifampicin	2µg/ml
2	Ethambutol	2.5µg/ml
3	Streptomycin	2µg/ml
4	Isoniazid	0.1µg/ml
5	Pyrazinamide	100µg/ml
6	Ofloxacin	2µg/ml
7	Amikacin	1µg/ml
8	Kanamycin	5µg/ml
9	Ethionamide	1.25µg/ml
10	Cycloserine	80µg/ml
11	Capreomycin	2.5µg/ml

Evaluation of Mycobacterium culture by Fluorescence method:

Concentrated sediment was inoculated in MGIT 960 medium and incubated at 37^{0}C. The MGIT 960 TB medium is an enriched Middlebrook 7H9 base. The MGIT tubes contain an oxygen quenched flurochrome (tris 4, 7-diphenyl-1, 10-phenonthroline ruthenium chloride pentahydrate) embedded in silicon at the bottom of the tube. During bacterial growth in the tube, the free oxygen is utilized and is replaced with carbon dioxide. With depletion of free oxygen, the fluorochrome is no longer inhibited, resulting in fluorescence within the MGIT tube when visualized under UV light. The intensity of fluorescence is directly proportional to

the extent of oxygen depletion. When a tube beeps in the instrument, it indicates the growth of Mycobacteria in the medium. The contamination can be ruled out by using blood agar and the sterility and positivity checked by ZN smear microscopy. PNB test (para-nitro benzoic acid) is used to differentiate between *Mycobacterium tuberculosis* complex and NTM.

Drug susceptibility Testing (DST):

Stock drug solutions were made as follows; Ethambutol, Streptomycin, Isoniazid, were dissolved in sterile double distilled water (DDW). Rifampicin was dissolved in N, N-Dimethylformamide (DMFO). These stock solutions were filter-sterilized and stored frozen at -20°C. Before the drug susceptibility testing, a tube of each stock solution of drug was thawed and diluted to the requisite concentration; added to each 7H11 vial with a tuberculin syringe, one vial being used for each drug and one vial without drug to serve as a control. 0.1 ml of culture was inoculated into each of the drug-containing vial. For the control inoculums, 0.1 ml of this suspension was added to 9.9 ml of diluents (DDW). After thorough mixing, 0.1 ml of this was inoculated into the control vial. The inoculation of this 1:100 dilution of the suspension into the control was done to determine the conventional 1% level of resistance by comparing the growth in the control and drug-containing vials[5,6].

**Drug**	**MIC**
Rifampicin	1.0µg/ml
Ethambutol	5.0µg/ml
Streptomycin	1.0µg/ml
Isoniazid	0.1µg/ml

Supplementary Table 1. Primary Reasons for not completing the treatment and exclusion from the final analysis.

Withdrawal Reason	No. of Patients	MIP arm Total recruited n=449	%	Placebo Arm Total recruited n=441	%
Death	20	12	2.67	8	1.81
Unwilling	79	39	8.68	40	9.07
Defaulter	94	42	9.35	52	11.79
Pregnancy	4	1	0.22	3	0.68
PI Decision	11	6	1.34	5	1.13
Protocol Violations	40	21	4.67	19	4.31
Total Number of patients not completing the treatment	248	121	26.94	127	28.79

Supplementary Table 2. Baseline features of the patients who did not complete the treatment and were not included in the Per Protocol analysis

Characteristic(s)	Patients in MIP Arm, Who did not complete the treatment (N=121) n (%)	Patients in placebo Arm, Who did not complete the treatment (N=127)	p Value	Odds Ratio	95% Confidence Interval
Age Group					
18-30 years	54 (44.6%)	58 (45.7%)	0.87	0.96	0.56-1.63
31-44 years	41 (33.9%)	48 (37.8%)	0.52	0.83	0.48-1.47
≥ 45 years	26 (21.5%)	21 (16.5%)	0.32	1.38	0.69-2.76
Gender					
Male	92(76.03%)	101 (79.53%)	0.2	0.68	0.36-1.29
Female	29(23.97%)	26 (20.47%)	0.5	1.2	0.64-2.34
BMI					
< 18.5	119 (98.35%)	121 (95.28%)	0.15	2.55	0.89-7.28
≥ 18.5	02 (1.65%)	6 (4.72%)	0.15	0.39	0.14-1.11
Reason for Inclusion in CAT-II					
Treatment Failure in CAT-I	11 (9.1%)	13 (10.24%)	0.76	0.87	0.34-2.22
Treatment after Default	60(49.58%)	49 (38.58%)	0.08	1.56	0.92-2.68
Relapse	50 (41.32%)	65 (51.18%)	0.12	0.67	0.39-1.14
Resistance to Drugs at Treatment Initiation					
> 3 drugs	0	0			
2-3 drugs	25 (20.66%)	27 (21.26)	0.91	0.96	0.5-1.86
1 drug	30 (24.8%)	16 (12.6%)	0.014	2.28	1.12-4.77
Streptomycin	16 (13.22%)	19 (14.96%)	0.69	0.87	0.39-1.88
Isoniazid	35 (28.93%)	32(25.2%)	0.5	1.2	0.66-2.2

Rifampicin<br>Ethambutol<br>Pyrazinamide	07(5.79%)<br>22 (18.2%)	5 (3.94%)<br>18 (14.2%)<br>ND	0.5<br>0.39	1.5<br>1.35	0.39-6.15<br>0.65-2.83
Sputum AFB Smear Grade					
1+	41 (33.9%)	39 (30.7%)	0.59	1.16	0.65-2.04
2+	24 (19.8%)	40 (31.5%)	0.036	0.54	0.29-1.0
3+	53 (43.8%)	42 (33.1%)	0.08	1.58	0.91-2.7
Sc	3 (2.5%)	6 (4.7%)	0.27	0.55	0.22-1.4
Chest Radiography at Treatment Initiation					
Bilateral cavitations	105 (86.8%)	103 (81.1%)	0.22	1.53	0.73-3.23
Unilateral cavitations	16 (13.2%)	24 (18.9%)	0.22	0.65	0.31-1.37
No cavitations	-	-			
Radiographic Severity of Disease at Initiation‡					
Minimal	14 (11.8%)	17 (13.4%)	0.68	0.85	0.37-1.95
Moderately Advanced	78 (64.5%)	84 (66.14%)	0.85	0.95	0.54-1.66
Far Advanced	28 (23.1%)	26 (20.47%)	0.59	1.18	0.62-2.26

Supplementary Table 3. Cure and effect size- in Modified ITT and Per Protocol at the end of 39th week.

	MIP Arm		Placebo Arm		p Value	Effect Size (95% CI)
	n	Cure (%)	n	Cure (%)		
Modified ITT basis (n= 890)	449	309 (68.8%)	441	284 (64.4%)	0.162	4.4% (-1.7% to 10.6%)
Per Protocol basis (n = 642)	328	309 (94.2%)	314	284 (90.4%)	0.073	4.2% (-0.41% to 7.81%)

Supplementary Table 4. Non-vaccine related adverse events observed in the two groups

Sl. No.	System/Event Category	MIP Arm (N = 449)	Placebo Arm (N=441)
1	Gastrointestinal	44	73
2	Haematological	2	4
3	CNS	-	2
4	Renal	1	-
5	CVS	10	13
6	Respiratory	17	11
7	Skin	11	17
8	Hepatic	3	6
9	Tuberculosis Related	17	26
10	Parasthesia	-	1
11	Arthalgia	-	1
12	Lymphadenitis	2	-
13	General	52	63
	**Total**	**159**	**217**

Supplementary Table 5. Sub group analysis of baseline characteristics of cured patients included in the Modified ITT

Characteristic(s)	All patients in MIP Arm, n (%) (N= 449)	Cured in MIP Arm, n (% of the subgroup) (N= 309)	All patients in Placebo Arm, n (%) (N= 441)	Cured in Placebo Arm, n (% of the subgroup) (N= 284)	p Value	Odds Ratio	95% Confidence Interval
Age Group							
18-30 years	249 (55.5%)	186(74.7%)	224 (50.8%)	152 (67.8%)	0.1	1.4	0.92-2.13
31-44 years	133 (29.6%)	85 (63.9%)	154 (34.9%)	93 (60.4%)	0.54	1.16	0.7-1.93
≥ 45 years	67 (14.9%)	38 (56.7%)	63 (14.3%)	39 (61.9%)	0.547	0.8	0.38-1.72
Gender							
Male	317 (70.6%)	211(66.6%)	324 (73.5%)	199 (61.4%)	0.175	1.25	0.89-1.75
Female	132 (29.4%)	98 (74.2%)	117 (26.5%)	85 (72.6%)	0.776	1.08	0.59-1.98
BMI							
< 18.5	441 (98.2%)	303(68.7%)	425 (96.4%)	274 (64.5%)	0.186	1.21	0.9-1.62
≥ 18.5	8(1.8%)	6 (75%)	16 (3.6%)	10 (62.5%)	0.45	1.8	0.21-23.36
Reason for Inclusion in CAT-II							
Treatment Failure in CAT-I	34 (7.6%)	21 (61.8%)	24 (5.4%)	8 (33.3%)	0.033*	3.23	0.95-11.25
Treatment after Default	189 (42.1%)	122 (64.5%)	174 (39.5%)	115 (66.1%)	0.758	0.93	0.59-1.47
Relapse	226 (50.3%)	166(73.5%)	243 (55.1%)	161 (66.3%)	0.090	1.4	0.93-2.14

Resistance to Drugs at Treatment Initiation							
> 3 drugs	0	0	0	0			
2-3 drugs	41 (9.1%)	16 (39%)	48 (10.9%)	16 (33.3%)	0.577	1.28	0.49-3.33
1 drug	84 (18.7%)	54 (64.3%)	60 (13.6%)	36 (60%)	0.6	1.2	0.57-2.51
Streptomycin	40 (8.9%)	23 (57.5%)	44 (10%)	18 (40.9%)	0.129	1.95	0.75-5.1
Isoniazid	78 (17.4%)	41 (52.6%)	80 (18.1%)	40 (50%)	0.747	1.1	0.57-2.17
Rifampicin	15 (3.3%)	8 (53.3%)	9 (2%)	3 (33.3%)	0.3	2.28	0.32-19.1
Ethambutol	40 (8.9%)	18 (45%)	30 (6.8%)	10 (33.3%)	0.324	1.64	0.55-4.94
Sputum AFB Smear Grade							
1+	145 (32.3%)	99 (68.3%)	122 (27.7%)	78 (63.9%)	0.455	1.21	0.71-2.08
2+	117 (26.1%)	87 (74.4%)	127 (28.8%)	83 (65.4%)	0.126	1.54	0.85-2.78
3+	168 (37.4%)	107 63.7%)	170 (38.5%)	108 (63.5%)	0.975	1.0	0.63-1.61
Sc	19 (4.2%)	16 (84.2%)	22 (5%)	15 (68.2%)	0.21	2.28	0.82-6.34
Chest Radiography at Treatment Initiation	353 (78.6%)	235(66.6%)	336 (76.2%)	208 (61.9%)	0.201	1.22	0.87-1.69
Bilateral cavitations	96 (21.4%)	74 (77.1%)	105 (23.8%)	76 (72.4%)	0.444	1.28	0.65-2.57
Unilateral cavitations	-	-	-	-			
No cavitations							

Radiographic Severity of Disease at Initiation‡							
Minimal	54 (12%)	40 (74.1%)	57 (12.9%)	38 (66.6%)	0.393	1.43	0.58-3.55
Moderately Advanced	306 (68.2%)	214 69.9%)	304 (68.9%)	201 (66.1%)	0.312	1.19	0.84-1.7
Far Advanced	88 (19·6%)	55 (62.5%)	80 (18.1%)	45 (56.3%)	0.410	1.29	0.67-2.52

‡ Radiographic severity at baseline not available for 1 patient; * Significant

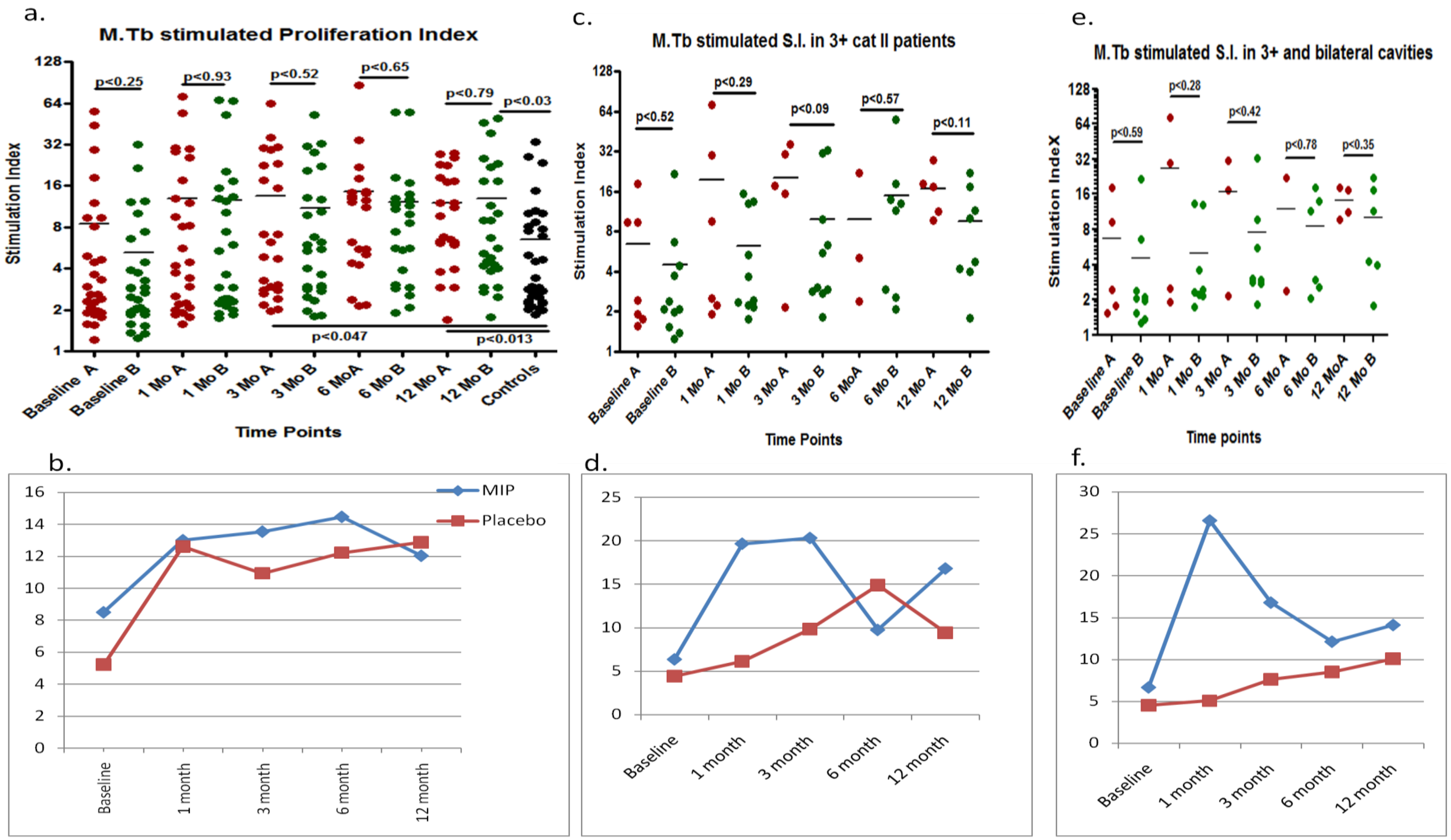

Supplementary Figure 1. Proliferation indices of Peripheral blood mononuclear cells (PBMCs) on ex-vivo re-stimulation with *M.Tb* and **a.** Scatter-graph of stimulation indices in two groups (A= MIP and B = Placebo) of patients re-stimulated with *M. Tb*. at different time points. **b.** Mean stimulation indices of scatter-graph shown in a. **c.** Scatter-graph of stimulation indices in two groups of patients with AFB 3+ **d.** Mean stimulation indices of scatter-graph shown in **c. e.** Scatter-graph of stimulation indices in two groups of patients with AFB3+ and bilateral cavities. **f.** Mean stimulation indices of scatter-graph shown in **e.**

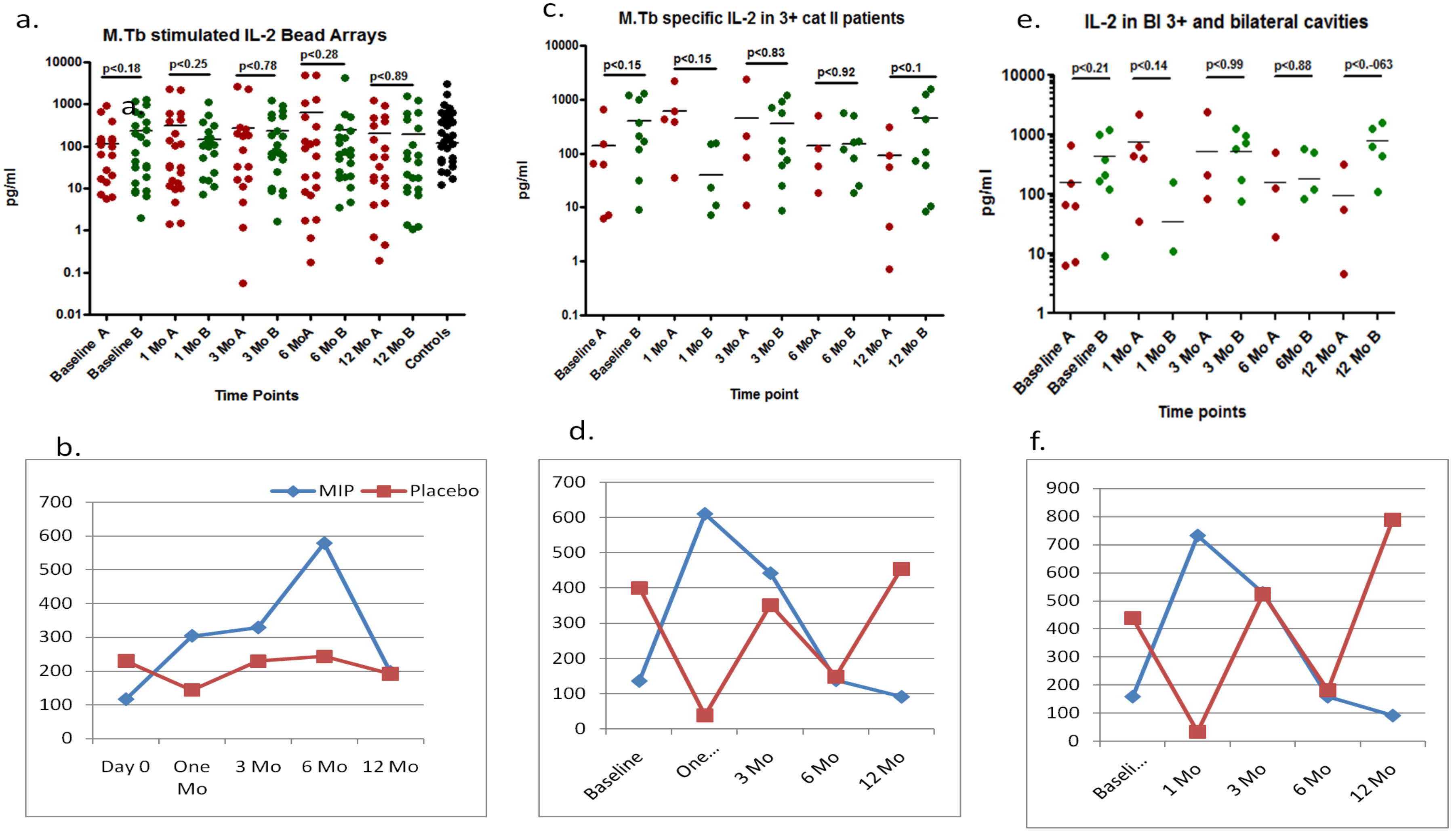

Supplementary Figure 2. Scatter-graph of amount IL-2 produced in the supernatant in pg/ml using multiplex bead arrays after stimulation of PBMCs with *M.tb* . **a.** Scatter-graph of IL-2 secretion in two groups (A= MIP and B = Placebo) of patients re-stimulated with *M. Tb*. at different time points. **b**. Mean IL-2 production MIP and Placebo groups. **c.** Scatter-graph of IL-2 production two groups of patients with AFB 3+ **d.** Mean IL-2 production of scatter-graph shown in **c. e.** Scatter-graph of IL-2 in two groups of patients with AFB3+ and bilateral cavities. **f.** Mean IL-2 amounts of scatter-graph shown in **e**.

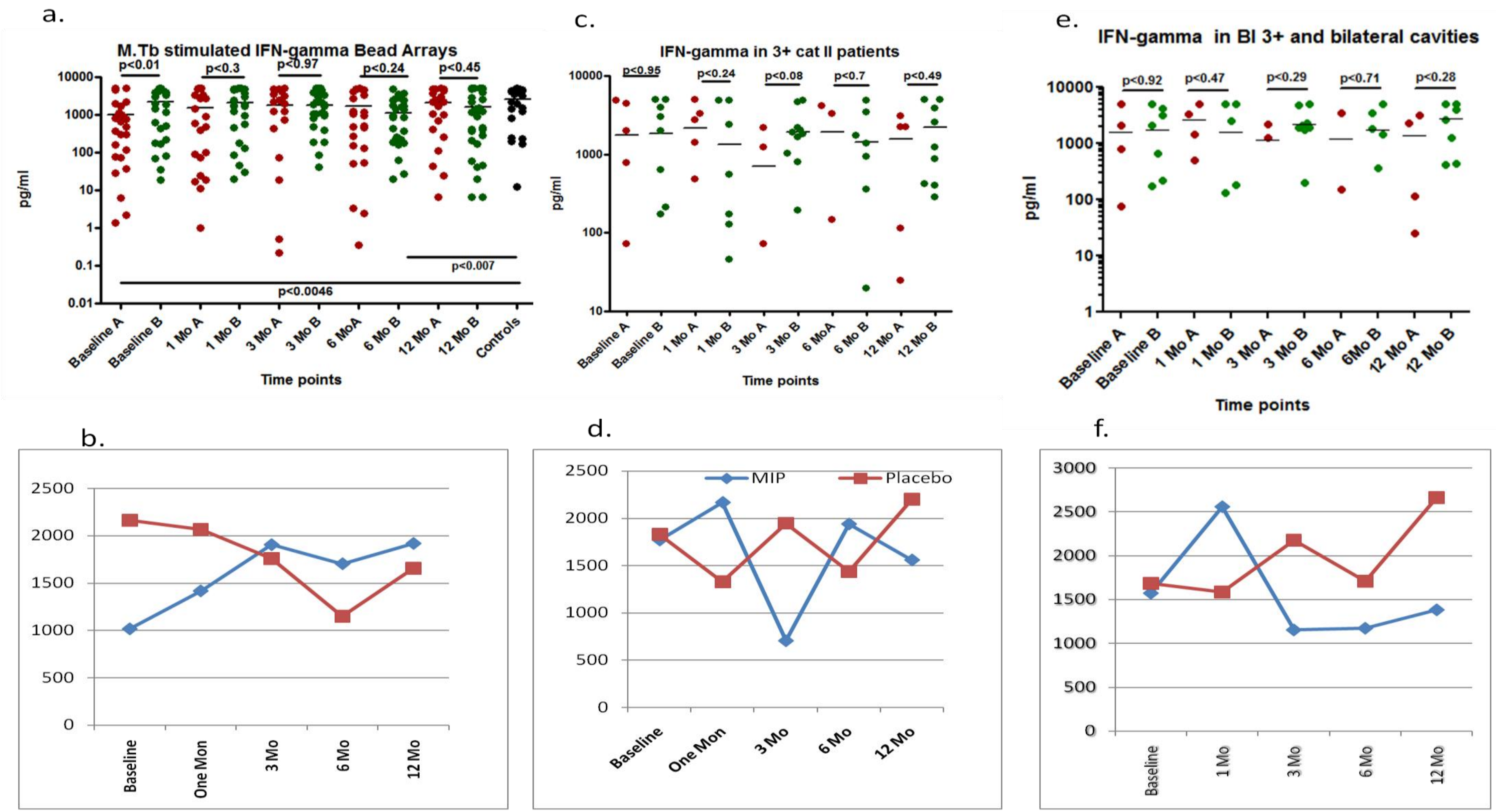

Supplementary Figure 3. Scatter-graph of amount IFN-γ produced in the supernatant in pg/ml using multiplex bead arrays after stimulation of PBMCs with *M.tb* . **a.** Scatter-graph of IFN-γ secretion in two groups (A= MIP and B = Placebo) of patients re-stimulated with *M. Tb*. at different time points. **b**. Mean IFN-γ production MIP and Placebo groups. **c.** Scatter-graph of IFN-γ production two groups of patients with AFB 3+ **d.** Mean IFN-γ production of scatter-graph shown in **c. e.** Scatter-graph of IFN-γ in two groups of patients with AFB3+ and bilateral cavities. **f.** Mean IFN-γ amounts of scatter-graph shown in **e**.

References:

1. Mathew, P., Kuo, Y. H., Vazirani, B., Eng, R. H. & Weinstein, M. P. Are three sputum acid-fast bacillus smears necessary for discontinuing tuberculosis isolation? *J Clin Microbiol* **40**, 3482-4 (2002).
2. Sula, L. & Langerova, M. Drug Sensitivity-Resistance Determination and Simple Enzymatic Tests for the Differentiation of Mycobacteria. *Bull World Health Organ* **29**, 579-88 (1963).
3. Canetti, G. et al. Mycobacteria: Laboratory Methods for Testing Drug Sensitivity and Resistance. *Bull World Health Organ* **29**, 565-78 (1963).
4. Leitritz, L. et al. Evaluation of BACTEC MGIT 960 and BACTEC 460TB systems for recovery of mycobacteria from clinical specimens of a university hospital with low incidence of tuberculosis. *J Clin Microbiol* **39**, 3764-7 (2001).
5. Rusch-Gerdes, S. et al. Multicenter evaluation of the mycobacteria growth indicator tube for testing susceptibility of Mycobacterium tuberculosis to first-line drugs. *J Clin Microbiol* **37**, 45-8 (1999).
6. Rusch-Gerdes, S., Pfyffer, G. E., Casal, M., Chadwick, M. & Siddiqi, S. Multicenter laboratory validation of the BACTEC MGIT 960 technique for testing susceptibilities of Mycobacterium tuberculosis to classical second-line drugs and newer antimicrobials. *J Clin Microbiol* **44**, 688-92 (2006).
